# International Practices of Speech‐Language Pathologists Working with Bilingual Speakers with Primary Progressive Aphasia

**DOI:** 10.1002/alz.093450

**Published:** 2025-01-09

**Authors:** Stephanie M Grasso, Jeanne Gallée, Jade Cartwright, Regina Jokel, Monica Lavoie, Ellen McGowan, Margaret Pozzebon, Bárbara Costa Beber, Guillaume Duboisdindien, Núria Montagut, Monica Norvik, Taiki Sugimoto, Rosemary Townsend, Nina Unger, Ingvild E. Wisnes E. Wisnes, Anna Volkmer

**Affiliations:** ^1^ University of Texas, Austin, TX USA; ^2^ University of Washington School of Medicine, Seattle, WA USA; ^3^ University of Tasmania, Tasmania Australia; ^4^ Baycrest Health Sceinces, Toronto, ON Canada; ^5^ Clinique Interdisciplinaire de Mémoire, CHU de Québec‐Université Laval, Quebec, QC Canada; ^6^ Salford University, Salford, Greater Manchester United Kingdom; ^7^ margaret.pozzebon@iinet.net.au, Melbourne, VIC Australia; ^8^ Universidade Federal de Ciências da Saúde de Porto Alegre (UFCSPA), Porto Alegre, RS Brazil; ^9^ Laval University, Quebec City, QC Canada; ^10^ Alzheimer’s disease and other cognitive disorders Unit. Hospital Clínic. Fundació Clínic per a la Recerca Biomèdica, IDIBAPS, Universitat de Barcelona, Barcelona Spain; ^11^ Norwegian University of Science and Technology, Trondheim, Sør‐Trøndelag Norway; ^12^ University of Washington, Seattle, WA USA; ^13^ Dyscovery Aphasia, Leatherhead, Surrey United Kingdom; ^14^ Universitätsmedizin Greifswald, Mecklenburg‐Western Pomerania Germany; ^15^ University of Oslo, Oslo Norway; ^16^ Department of Psychology & Language Sciences, University College London, London United Kingdom

## Abstract

**Background:**

Although primary progressive aphasia (PPA) is considered a rarer form of dementia, individuals living with PPA are increasingly identified by healthcare professionals. Research investigating speech‐language assessment and intervention in PPA has been conducted primarily in monolingual speakers and little is known about clinical decision‐making of speech‐language pathologists (SLPs) working with bilinguals with PPA.

**Methods:**

A comprehensive survey containing questions regarding clinician confidence, prioritization, and ratings of basic competency for Volkmer, Cartwright, Ruggero et al.’s (2023) best practice principles was constructed with questions that also queried practices pertaining to working with bilingual populations. Data was collected anonymously, via the Qualtrics survey platform and the survey was disseminated via social media and through social networks of study team members.

**Results:**

A total of 185 participants responded with representation from 27 countries. In total, bilingual participants spoke a total of 39 different languages. The average number of languages spoken by respondents was 1.86 (SD = 1.08). Twenty‐three percent of respondents reported that they provided clinical services bilingually and 28% identified as bicultural. Respondents indicated that coursework in their training to become SLPs related to bilingual neurogenic communication disorders was covered for less than two hours (39%), less than five hours (33%), or more than five hours (28%). The majority of respondents indicated that they sometimes or typically worked with an interpreter or translator for conducting bilingual assessments (43%) or performed them independently (18%). When asked which language respondents typically assess participants in, 5% indicated the maternal language, 39% indicated the dominant/most functional language(s), and 54% indicated the language(s) the clinician felt comfortable speaking.

**Conclusion:**

This study reports, for the first time, the practices of speech‐language pathologists working with bilingual speakers with PPA. Results indicate that SLPs are likely to receive some exposure to bilingual adult neurogenic communication disorders in their training. SLPs are more likely to assess bilingual individuals with PPA in the languages clinicians speak, or in the participant’s most functional/dominant language(s). Additional international forums are needed to extend core principles and philosophies of SLP practices, particularly when individuals living with PPA speak more than one language.

